# Plant-based ink properties and storage stability for inkjet printing

**DOI:** 10.1007/s11356-023-31714-y

**Published:** 2024-01-04

**Authors:** Alka Madhukar Thakker, Danmei Sun

**Affiliations:** School of Textiles and Design, Heriot-Watt University, Galashiels, TD1 3HF UK

**Keywords:** Plant-based inks, Digital printing, Viscosity, Surface tension, Conductivity, Coffee ring effect

## Abstract

In this sustainability-oriented research, the properties of plant-based inks were evaluated denoting the viscosity range of 8.5 to 10 cP, the relative density of 1.06, conductivity value of 2.51 mS/cm, and the surface tension of 60 mN/m and pH of 4.9 to be most effective for inkjet printing. The changes in these properties to the one-month storage phase are detailed as determined with attenuated total reflectance – Fourier transform infrared spectroscopy, viscometer, and tensiometer. The varied colours of plant-based inks were stable to storage time except for the blue colour ink made from bio indigo herb that displayed agal-like sediments. After the storage phase, the plant-based inks exhibited anti-thixotropic viscosity except for yellow colour plant-based ink demonstrating thixotropic behaviour. High conductivity values of 18.5 and 15.6 mS/Cm were noted for blue and black colour plant-based inks, indicating their potential for constituting conducting inks; however, the conductivity values dropped to 7.5 and 9.5, respectively, after 1 month. The pH and surface tension were found steady during the storage period. The study of the life cycle analysis of plant-based inks is suggested for future work. The significance of this work in developing plant-based inks for inkjet printing of textiles lies in the convergence of sustainability and innovation. Plant-based inks can offer an eco-friendly alternative to traditional synthetic inks that are used currently, which provides a knowledge base for good practises meeting the environmentally conscious in the digital printing of the textile industry. These developed inks from this study can not only reduce the environmental impact but also promote a healthier ecosystem.

## Introduction

The field of inkjet printing on textile substrates with plant-based colours is the least explored area. There exists insufficient literature on the experimentation of inkjet printing with plant-based ink properties. On the other hand, synthetic inks the market. Hence, the authors are formulating plant-based inks and testing their properties for application on fabrics, thereby attempting to resolve the environmental and human health hazard posed by petroleum-based artificial inks. Water-based polymers namely polyvinyl alcohol and waterborne polyester were utilised for dispersing dye ink formulation. All the ingredients indicated in Table 1 were taken by percentage weight (Gao, Xing, Hou, & Chen, 2019). The line concoction was homogenised by stirring at 1000 rounds per minute at 25 °C and vacuum filtered with a 0.22 μm membrane filter. The newly formulated disperse dye inks were assessed for their physical properties, for example, the properties of polyvinyl alcohol polymer-based disperse dye ink are given in Table 2 (Gao, Xing, Hou, & Chen, 2019). Excellent fastness properties were obtained. Higher colour values and colour saturation were gained. The highest K/S value of 3.88 and colour saturation value of 64.97 was noted on non-pre-treated polyester fabric inkjet printed with waterborne polyester-based disperse dye ink (Gao, Xing, Hou, & Chen, 2019). It is a concern that the constituted ink would contribute to emissions and effluents due to artificial solvents and additives.

**Table 1:** Water-based disperse dye ink formulation (Gao, Xing, Hou, & Chen, 2019).

Sample	Dye (wt%)	TD-1109 (wt%)	DEG (wt%)	Water-based polymers (wt%)	EG (wt%)	Organosilicon defoamer (wt%)	TEOA (wt%)	Water (wt%)
1-1	5.01	2.76	5.01	0.0	22.5	0.5	0.05	64.17
1-2a	5.01	2.76	5.01	0.5	17.5	0.5	0.05	68.67
1-3a	5.01	2.76	5.01	0.25	20.0	0.5	0.05	66.42
1-4a	5.01	2.76	5.01	1.0	12.5	0.5	0.05	73.17
1-5a	5.01	2.76	5.01	1.5	7.5	0.5	0.05	77.67
1-2b	5.01	2.76	5.01	0.5	17.5	0.5	0.05	68.67
1-3b	5.01	2.76	5.01	0.25	20.0	0.5	0.05	66.42
1-4b	5.01	2.76	5.01	1.0	12.5	0.5	0.05	73.17
1-5b	5.01	2.76	5.01	1.5	7.5	0.5	0.05	77.67

Note: Two water-based polymers are (a) polyvinyl alcohol and (b) waterborne polyester. TD is a hydrophilic polyacrylic acid block copolymer as a dispersing agent, DEG is diethylene glycol as a wetting agent, EG is ethylene glycol as a wetting agent, and TEOA is triethanolamine as a pH regulator

**Table 2:** Properties of water-based disperse dye inks (Gao, Xing, Hou, & Chen, 2019).

Inks	Viscosity (mPa s)	Conductivity (mS cm^−1^)	pH	Surface tension (mN m^−1^)	*Z*-Average size (nm)
1.1	2.60	0.58	8.28	31.79	153.6
1.2a	2.33	0.58	8.30	31.09	157.9
1.3a	2.78	0.57	8.23	33.28	166.7
1.4a	3.05	0.58	8.14	32.46	146.5
1.5a	3.24	0.58	8.20	32.22	161.7

Varied water-soluble alcohols such as diethylene glycol, triethylene glycol, 1,2-propanediol, 1,3-butanediol, glycerol, hexylene glycol, and isopropyl alcohol with different boiling points are 245.0, 288.3, 187.3, 209.0, 291.0, 197.0, and 83.0 correspondingly were employed for formulating inks for digital printing of cotton fabrics (Hou, Chen, Tieling, & Zhenzhen, 2019). It was observed that the inks with high boiling points proved as good humectants; however, storage stability and print quality were lower than inks formulated with low boiling points and vice versa. The fastness properties obtained on cotton fabrics inkjet printed with varied formulated inks were good to excellent for all the inks (Hou, Chen, Tieling, & Zhenzhen, 2019). A point to note is that inkjet inks are low viscosity fluids with a surprisingly simple composition consisting of a solvent and colourant as prime ingredients with other specific auxiliaries. It is imperative to keep the prime ingredients non-toxic as they would be handled in large quantities at a manufacturing scale (Christie, 2015). Therefore, colour researchers, manufacturers, and users must address the inventions so that they are safe from start to end for the environment and human health. The Registration, Evaluation, Authorization of Chemicals (REACH) aims to address the issue by imposing complete material and process transparency reinforced with life cycle analysis (LCA) of each chemical in the market (Christie, 2015). Table 3 summarises varied ink properties along with their purpose. The device to measure those properties and their units of measurement are also listed.

**Table 3:** Ink properties and the effect on inkjet printing (IMI Europe Limited, 2017a) (IMI Europe Limited, 2017b) (Encyclopædia Britannica, Inc., 2022a).

Physical property	Purpose and function	Instrument for measurement	Unit of measurement
Viscosity	Affects the drop formation and ink flow through the print head nozzle	Viscometer	Centipoise, cP
Density	Determines viscosity	Hydrometer	Gramme per cubic centimetre, g/cm^3^
Surface tension	Affects satellite formation and interaction with the substrate	Tensiometer	Millinewton per metre, mN/m
Conductivity	Facilitate drop placement at the right place on the substrate. Determines waveform influencing drop volume, velocity, and trajectory	Electrical conductivity metre	Millisiemens per centimetre, mS/cm
pH	Determines inks stability	Digital pH metre	Unitless

**Fig. 1:**
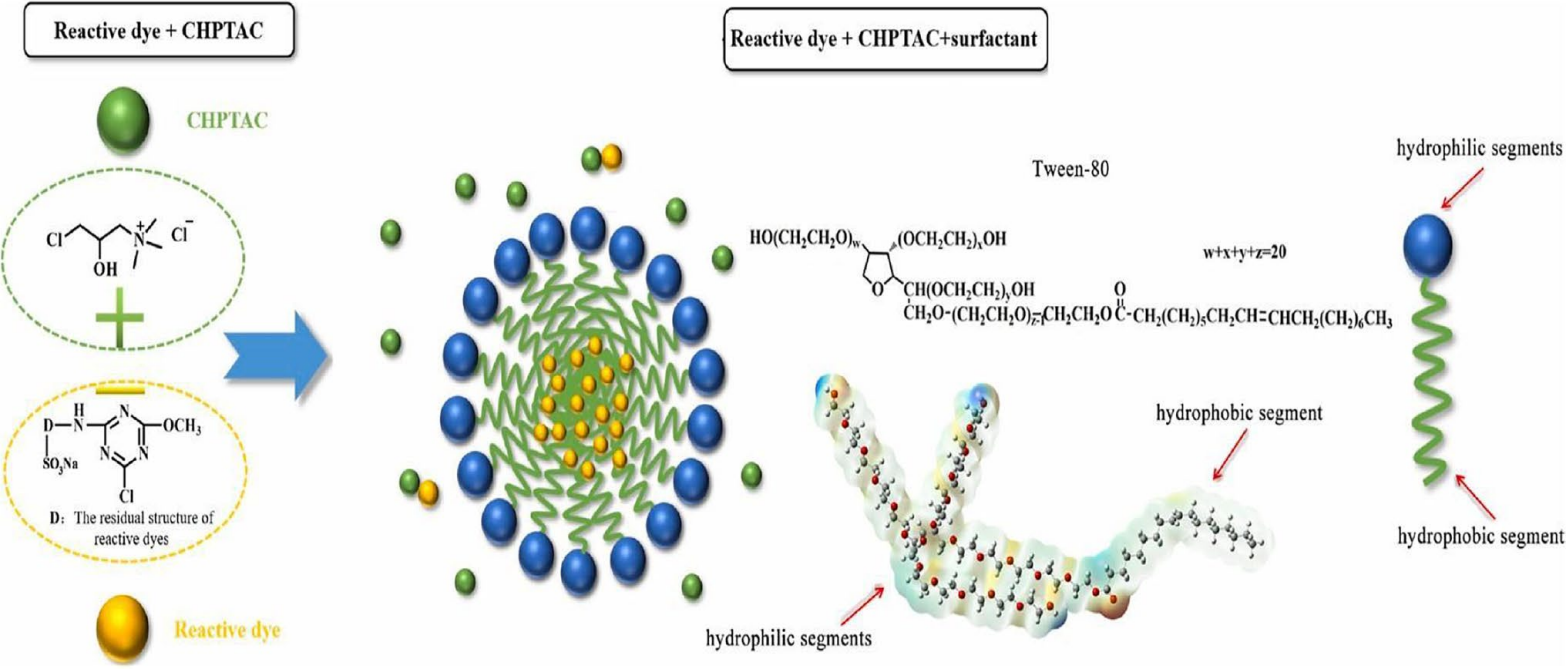
Hybrid ink model triad system consisting of reactive dye + 3-chloro-2-hydroxypropyl trimethyl ammonium chloride (CHPTAC) + Tween-80 surfactant (Wang, Yan, Hu, & Ji, 2020).

**Fig. 2:**
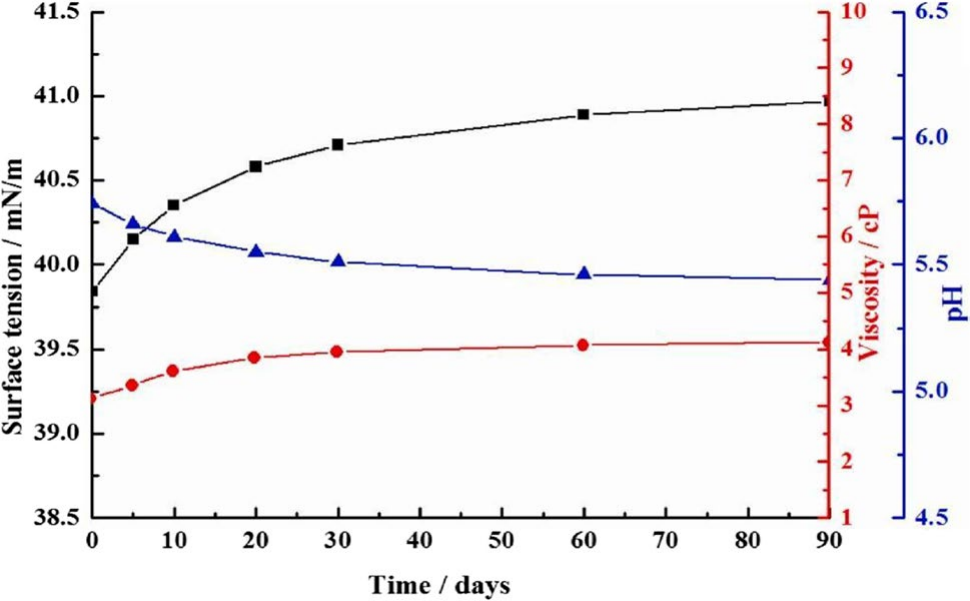
Hybrid ink stability to 3 months storage phase indicated with change in surface tension, pH, and viscosity (Wang, Yan, Hu, & Ji, 2020).

Synchronously, a stable ink was formulated with the fusion of reactive dye, 3-chloro-2-hydroxypropyl trimethyl ammonium chloride (CHPTAC), and Tween-80 surfactant as shown in Fig. 1. The K/S value obtained on cotton fabric inkjet printed with 30% CHPTAC was 21.98 and 16.02 without CHPTAC (Wang, Yan, Hu, & Ji, 2020). A newly formulated ink exhibited good stability to 90 days of storage period as indicated in Fig. 2, which demonstrates the changes in surface tension, pH, and viscosity of hybrid ink over a 3 months storage period. Additionally, it was noted that the K/S value acquired on cotton fabrics digitally printed with freshly prepared hybrid ink was 21.68. The K/S value noted on the cotton fabric inkjet printed with 3 months old hybrid ink was 21.33 indicating good stability of the ink (Wang, Yan, Hu, & Ji, 2020). This research aims to investigate plant-based ink properties and assess their stability during storage.

## Materials and methods

### Plant-based biomaterials

**Fig. 3:**
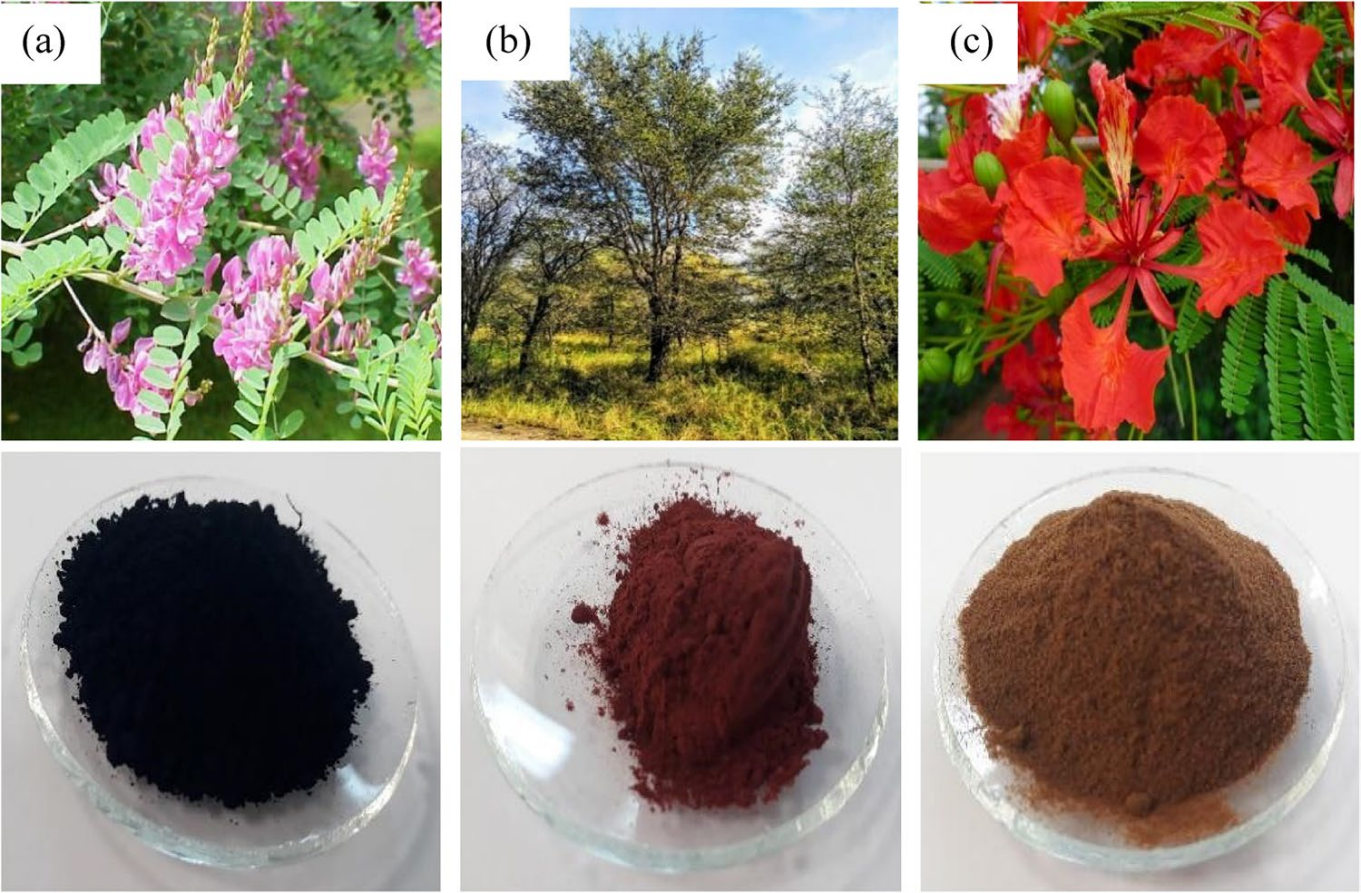
The plant materials selected to acquire C, M, Y, and K colours for inkjet printing. Top (green plant) and bottom (plant extracts). **a** Bio indigo leaves (C), **b** quebracho red bark (M), and **c** the flame of the forest flowers (Y), and K was obtained from the mixture of C, M, and Y plant materials (NatureLoc.com, 2022) (Oien, n.d.) (tannins.org, 2021).

The plant extracts utilised in the research were obtained from Ewe and Ply, Wonky Weaver, DT Craft and Design, & Wigwam Wool Work, UK, and Sodhani Biotech Pvt. Ltd., India. It was ensured that there were no endangered species among the selected plant materials. The plant materials are regrown and biodegradable hence sustainable, therefore, preferentially sourced for the study. Plant-based inks were formulated from the selected plant materials, as revealed in Fig. 3 following the recipe (stoichiometry and methodology) formulated by Thakker and Sun (Thakker, Sun, & Bucknall, 2022). The lab dips provided the colour values as illustrated in Tables 4 and 5. These colour values were utilised to develop a design, as illustrated in Fig. 3 in Adobe Photoshop 2021. The design represents solid areas and curves and straight lines suitable for print quality analysis. Logwood and the flame of the forest flowers extract materials were utilised to obtain yellow colour (Y) in formulating plant-based inks 1 and 2, respectively.

**Table 4:** Colour values of the formulated plant-based inks 1.

Plant extracts applied on cotton fabrics	L*	a*	b*	K/S
White cotton	96.54	3.08	−12.98	2.32
Bio indigo leaf extract (C)	43.31	−0.10	−16.25	10.561
Bio indigo leaf extract (LC)	56.79	0.40	−15.80	5.5805
Quebracho plant (M)	53.33	37.28	1.38	11.656
Quebracho plant (LM)	64.05	28.87	−3.51	6.5481
Logwood plant (Y)	12.05	39.29	20.76	16.377
Bio indigo leaf extract + quebracho plant + logwood plant (K)	8.40	34.86	14.47	27.159
Bio indigo leaf extract + quebracho plant + logwood plant (LK)	21.20	28.43	36.42	20.423
Bio indigo leaf extract + quebracho plant + logwood plant (LLK)	32.17	15.43	24.91	18.672

**Table 5:** Colour values of the formulated plant-based inks 2.

Plant extracts applied on cotton fabrics	L*	a*	b*	K/S
White cotton	94.73	3.17	−14.47	2.651
Bio indigo leaf extract (C)	65.45	−2.58	−23.53	17.152
Bio indigo leaf extract (LC)	83.64	−2.52	−12.70	5.4511
Quebracho plant (M)	34.44	48.51	6.79	18.121
Quebracho plant (LM)	46.14	39.94	2.88	6.2944
Sacred tree (Y)	65.94	9.46	63.45	10.186
Bio indigo leaf extract + quebracho plant + sacred tree plant (K)	47.59	7.67	25.40	16.034
Bio indigo leaf extract + quebracho plant + sacred tree plant (LK)	53.35	1.59	28.49	8.5952
Bio indigo leaf extract + quebracho plant + sacred tree plant (LLK)	53.67	0.14	28.10	4.9504

### Plant-based ink formulation

**Fig. 4:**
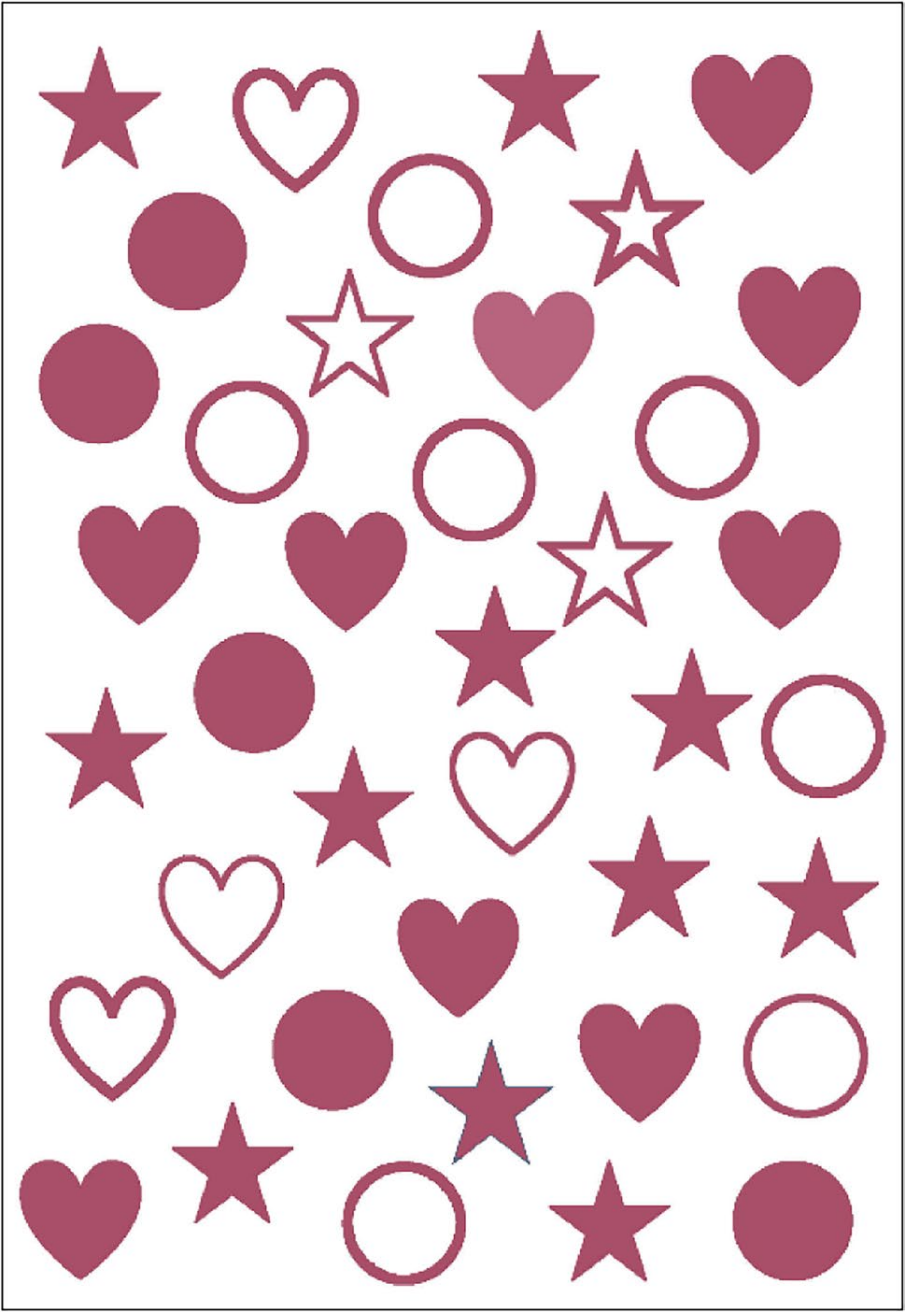
Design for inkjet printing on wool and cotton fabrics, Quebracho red colour.

The plant extract, distilled water, and glycerol were taken by weight when preparing plant-based ink ingredients. The procured plant extracts were commercially extracted from the Soxhlet apparatus and ball-milled, typically utilising distilled water as solvent. The plant extracts were water-soluble; still, the milling was performed with hand pestle mortar. It was done to facilitate the dispersion of plant extract powder into distilled water. The milled plant extract was gradually added to the distilled water and simultaneously stirred on a magnetic stirrer at elevated speed for 2 min. The stirred mixture was allowed to stand still for 1 h for the surplus plant extract particles to settle down. The mixture was strained and filtered using a 70 mm Whatman glass fibre filter to obtain a pure colour solution. The filter was changed in between to eliminate the sludge obtained. The weighed-up amount of glycerol was stirred into the filtered colour solution on a magnetic stirrer for 2 min at an elevated speed to obtain a homogenised solution. The resulting solution was filtered again through a microfilter (MF)—Millipore, 0.22 μm membrane (MCE) microfilter to ensure thorough filtration and homogenisation of the colloidal solutions of plant-based inks. The sterile microfilters were 47 mm hydrophilic nanofilters. Thereafter, the double-filtered ink was transferred into a sterile, air-tight bottle, and labelled appropriately. The entire process was repeated to obtain eight inks based on the C, M, Y, and K colour model of the printer. When working with small quantities of inks for percolation, the larger type of vacuum filtration method was overlooked to prevent the potential risk of ink wastage. Also, the sludge formation during purification would inhibit the implementation of vacuum filtrations. Each of the synthesised inks was further examined for various rheological properties. The cotton fabric was dip-dyed with formulated inks PI1 and PI2 (Lab Dips for reference purposes only) Fig. 4. The L*, a*, b*, and K/S values were noted on Datacolor 600 as organised in Tables 4 and 5.

During the pilot study, several lap dips were taken with varied permutations and combinations of herbs and plant extracts to acquire the C, M, Y, and K theory-based colours. Among those, the selected two sets are given herein in Tables 4 and 5. The initial lap dips are for reference purposes only. It enables one to understand how diverse or parallel the colour values are with synthetic counters; and accordingly, further changes were made as indicated in Tables 4 and 5. The lab dips assist in visual and technical analysis of colour. Correspondingly, the following stoichiometry was gained as indicated in Table 6 and standardised to formulate inks as given in Table 8. The details on standardisation of each of the ink properties are demonstrated in the results and discussion section.

**Table 6:** Stoichiometry of plant-based inks to acquire C, M, Y, and K colours.

Plant extracts applied on cotton fabrics	Colour	Colourant (g)	Water (ml)	Glycerol (ml)	Viscosity (cp)
Bio indigo leaf extract	C	9	76	15	L
Bio indigo leaf extract	LC	6	74	20	M
Quebracho plant	M	6	69	25	H
Quebracho plant	LM	4	76	20	M
Sacred tree	K	6	79	15	L
Bio indigo leaf extract + quebracho plant + sacred tree plant	K	11	64	25	H
Bio indigo leaf extract + quebracho plant + sacred tree plant	LK	11	69	20	M
Bio indigo leaf extract + quebracho plant + sacred tree plant	LLK	11	74	15	L

### Viscosity measurement

Viscosity is the resistance of a fluid (liquid or gas) to a change in shape or movement of neighbouring portions relative to one another. Viscosity denotes opposition to flow. The reciprocal of the viscosity is called fluidity, a measure of the ease of flow. The dimensions of dynamic viscosity (DV) can be obtained by Eq. 1.

1 $$\textrm{DV}=\frac{F\times T}{A}$$

where *F* is the force in Newton, *T* is time in seconds, and *A* is the area in square metres (m^2^).

**Fig. 5:**
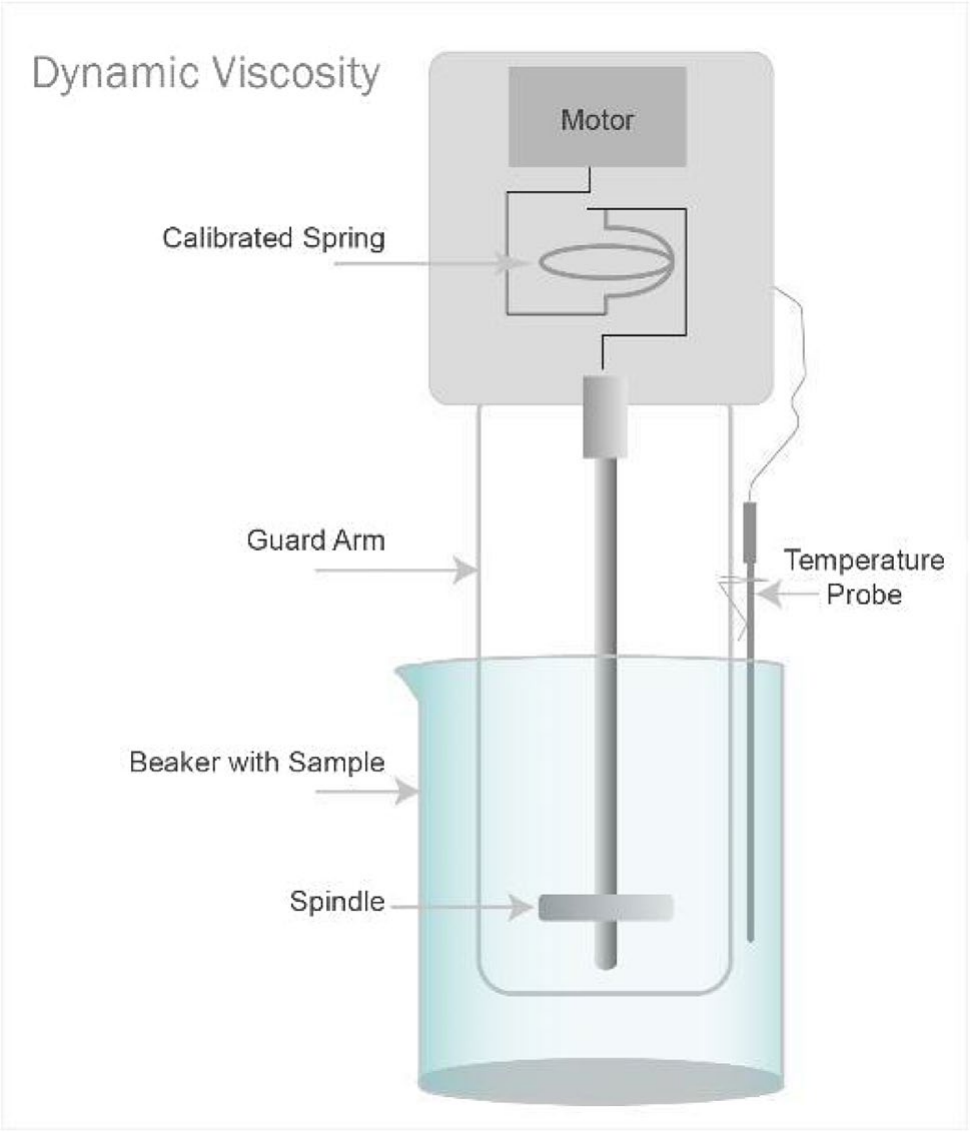
Viscometer in work (Particle Technology Labs, 2021).

**Fig. 6:**
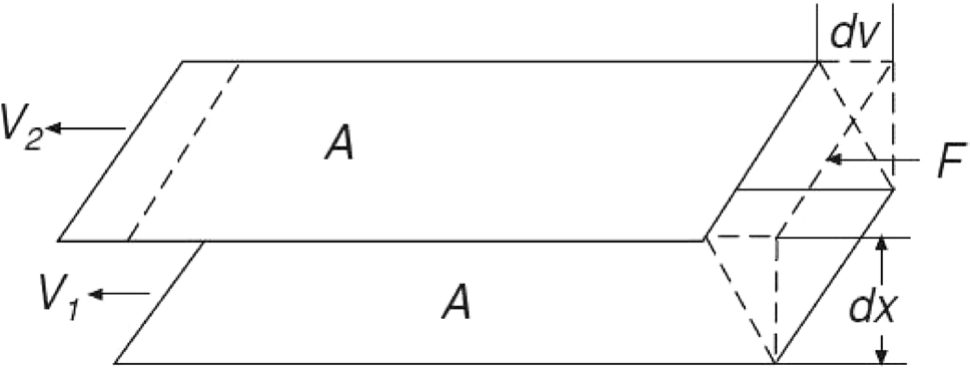
Viscosity defined (Particle Technology Labs, 2021).

As per the centimetre-gramme-second (CGS) systems of the unit, dynamic viscosity is denoted as centipoise (cP). It is the unit of dynamic viscosity (Marrion, 1994) (Merriam-Webster, Incorporated, 2022). Brookfield DV – II + Pro Viscometer could be utilised to measure the dynamic viscosity of the plant-based ink. The working principle is demonstrated in Fig. 5. The viscometer quantifies dynamic viscosity. The operating theory of a stand-alone rotational viscometer is to propel a spindle immersed in the fluid through a calibrated spring. The cylindrical spindle rotates at a well-defined speed, and the viscose tug of the fluid against the spindle causes spring deflection (Particle Technology Labs, 2021). The viscometer measures this resistance, and the digital reading is acquired in the unit of centipoise or “cP.” As force is applied to the fluid, it moves from one plane to another. The viscose fluid requires high shear force than the low viscose fluid. The two planes of fluids represented as dx turn in the same direction at two distinct velocities denoted as dv, as illustrated in Fig. 6 and Eqs. 2 and 3. The force required to maintain dv is in proportion to the velocity gradient. Therefore, the velocity gradient could be represented as demonstrated in Eq. 2 (Particle Technology Labs, 2021).

2 $$\textrm{Velocity}\ \textrm{gradient}=\frac{\textrm{dv}}{\textrm{dx}}$$

3 $$\frac{F}{A(T)}=\upeta \times \frac{dv}{dx}$$

where *F*/*A* is force per unit area, which is shear stress (*T*), Ƞ is viscosity, and dv/dx is the velocity gradient.

Interestingly, the velocity gradient denoted as dv/dx is the change in the speed of the multiple layers of fluid to each other, known as shear rate (*Y*). In other words, the shear rate is defined as the change of shear per unit time (Marrion, 1994). Eventually, viscosity η can be calculated by Eq. 4 (Particle Technology Labs, 2021).

4 $$\eta =\frac{T}{Y}\times 100$$

where *T* is shear stress, and *Y* is shear rate.

### The viscosity scale devised for the study

Based on the review of literature (Guo, 2012) (Mikkonen, 2017) (Tawiah et al., 2019), the following scale for viscosity was devised (Table 7).

**Table 7:** Viscosity scale.

Viscosity level	Viscosity range, cP
High (H)	>10 cP
Medium (M)	8.5–10
Low (L)	7–8.5

### Conductivity measurement

Electrical conductivity is the quality or power of conducting or transmitting electricity. Conductivity is measured in micro siemens per centimetre (μs/cm) or millisiemens per centimetre mS/cm (Merriam-Webster, Incorporated, 2022). Distilled water has a conductivity of 0.5 to 3 μmhos/cm (The United States Environment Protection Agency, 2012). The unit of electrical conductivity ρ is, by definition, the reciprocal of electrical resistivity, as signified in Eqs. 5 and 6 (getclac.com, 2021). Where *R* is resistance, *A* is an area of cross-section, and *L* is the measured length of a material.

5 $$\textrm{Resistivity}\ \left(\rho \right)=\frac{\textrm{RA}}{L}$$

6 $$\textrm{Conductivity}\ (C)=\frac{1}{\rho }$$

### Surface tension measurement

**Fig. 7:**
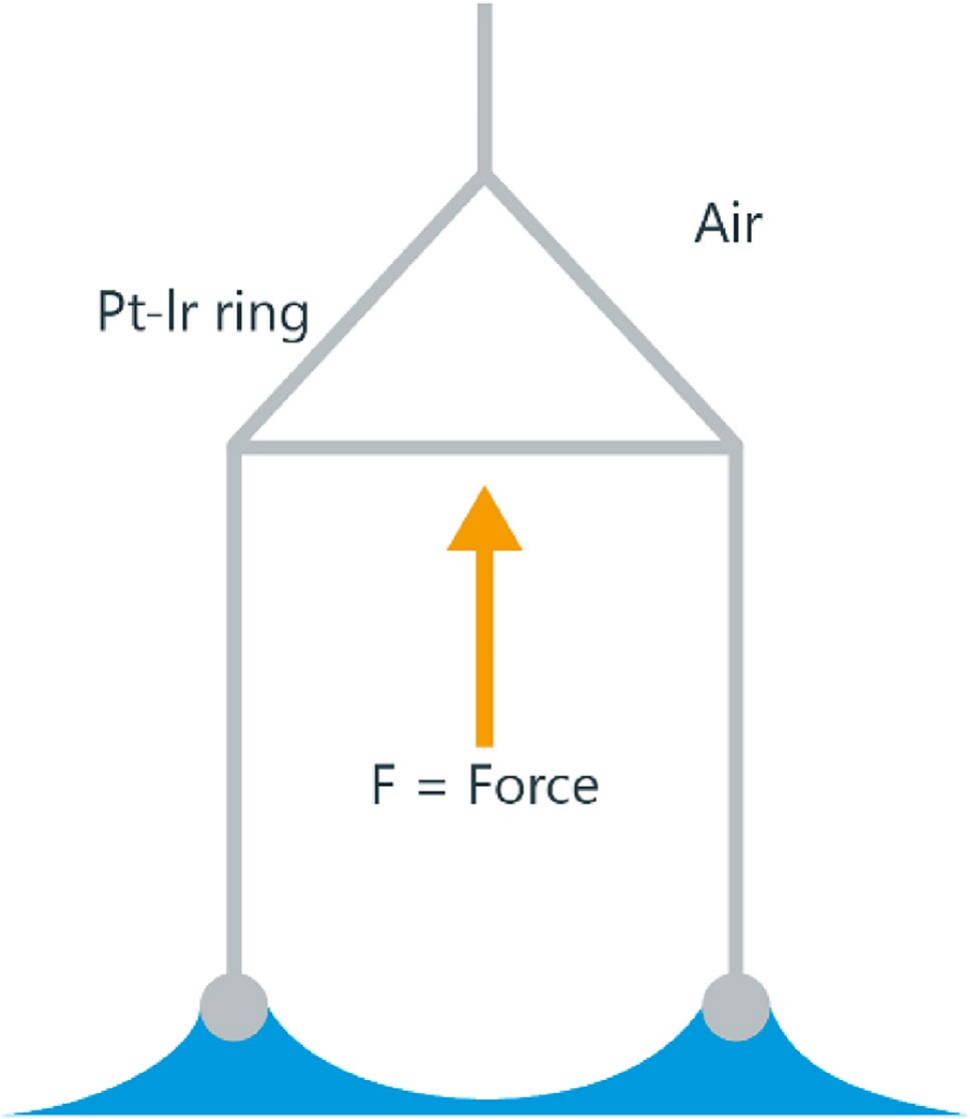
Du Noüy ring method (Kruss, n.d.-c).

Surface tension (Ƴ°) of a liquid is the attractive force exerted upon its surface molecules by the molecules beneath that tends to draw the surface molecules into the bulk of the liquid and makes the liquid assume the shape having the least surface area (Shaw, 1980). Surface tension may therefore be expressed in units of energy (joules) per unit area (square metres) or milliNewtons per metre (mN/m). The force tensiometer works on the principle of the ring-tear-off method (Kruss, n.d.-a), which is a precursor of the Du Noüy ring method (Kruss, n.d.-b), as shown in Fig. 7 and Eq. 7.

7 $$\sigma =\frac{F}{L\times \textrm{COS}\theta }$$

where *σ* is surface tension, *θ* is contact angle, *F* is maximum force, and *L* is wetted length.

The maximum force (*F*_max_) occurs when the lamella detaches and aligns vertically to the ring plane, as explained in Fig. 4. The wetted length of the ring is the sum of the outer and inner circumference. The platinum-iridium ring is utilised as it is inert and highly wettable, forming a contact angle of *θ* = 0°. Consequently, the Cos θ is 1 for liquids (Kruss, n.d.-d), as depicted in Eq. 7. Herein, the plant-based ink properties will be assessed by employing the above theories.

It is significant to note that in research, the amount of extract is concentrated in plant-based ink formulation; hence, significantly less quantity is consumed for inkjet printing than for dyeing. Consequently, it would defend agricultural land from being jeopardised for cultivating plants for fabric dyeing. The practical implication of this experiential research is that it would save both water and plants, as a result, propel climate action. Before this study, evidence of digital printing with plant-based formulations was purely anecdotal.

### Particle size and solubility

**Fig. 8:**
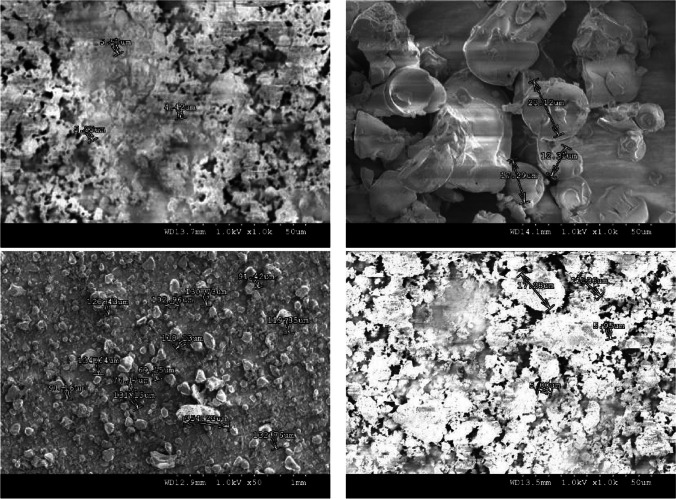
Particle size determined on SEM for **a** C, **b** M, **c** Y, and **d** K plant-based colours.

The particle size of plant extract would affect the solubility of plant extract in distilled water. Therefore, it is crucial to investigate the particle size and the solubility of plant-based ingredients while formulating inks. The plant-based colourant particles were analysed by a Hitachi S-4300 FE-SEM, field emission scanning electron microscope (FE-SEM). The computer connected to the SEM machine was switched ON, before the SEM machine was flashed before the test. The samples prepared beforehand were placed onto the stub with a tab to secure them. For analysis, the samples were attached to a rod with a knob that further carries the sample stage to the source of electrons. When the high voltage mode was ON, electrons were generated in the sample test chamber and images of samples were created that can be viewed in the computer. Figure 8 shows the images and particle sizes of C, M, Y, and K colourants, respectively.

The particle sizes of 2.03 μm, 17.57 μm, 91.85 μm, and 9.89 μm were noted for C, M, Y, and K colour plant materials, respectively, the ideal being 1 micron as recommended in the literature. However, since the plant extracts utilised for experimentation are water-soluble and plant-based ink formulations are double-filtered, the difference in particle size would dissipate and therefore would not block print jets. Further, the solute, a plant extract colour, when dissolved in distilled water (DW) as the solvent, forms a plant-based solution. Since the selected plant materials are known to be water-soluble, only the basic solubility of plant materials was determined in grammes per 100 grammes of solvent following Eq. 8 (Aus-e-tute, n.d.) (Leaf Group Ltd., 2018). Three variations of plant-based solutions and their respective solubility are given in Table 8.

**Table 8:** Plant-based inks recipe, viscosity variations, and solubility in distilled water.

Viscosity level	Colourant (g)	DW (%)	Glycerol (%)	Solubility (mg/ml)	Solubility scale
High (H)	15	55	30	27.27	Soluble
Medium (M)	15	65	20	23.07	Soluble
Low (L)	15	75	10	20	Soluble

8 $$\textrm{Solubility}\ \left(\%\right)=\frac{\textrm{Mass}\ \textrm{of}\ \textrm{solute}}{\textrm{Mass}\ \textrm{of}\ \textrm{solvent}}\times 100$$

## Results and discussion

### Appraisal of plant-based ink formulation

Initially, two sets of plant-based inks designated as PI1 and PI2 were constituted to perform research experiments and to analyse varied test parameters. The details are comprehended herein. The black plant-based black ink was achieved with a primary colour scheme rather than the complementary colour scheme.

#### Rheology of plant-based inks

The physical properties of the formulated plant-based inks were mathematically plotted and investigated. It is vital to note that the plant-based ink for the black colour is formulated from a mixture of the primary colours, namely, yellow + red + blue. The sources of yellow colour in PI1 and PI2 are the logwood plant and the flame of the forest flowers, respectively. Therefore, the consequent changes in rheology were systematically documented and investigated.

#### Relative density

**Fig. 9:**
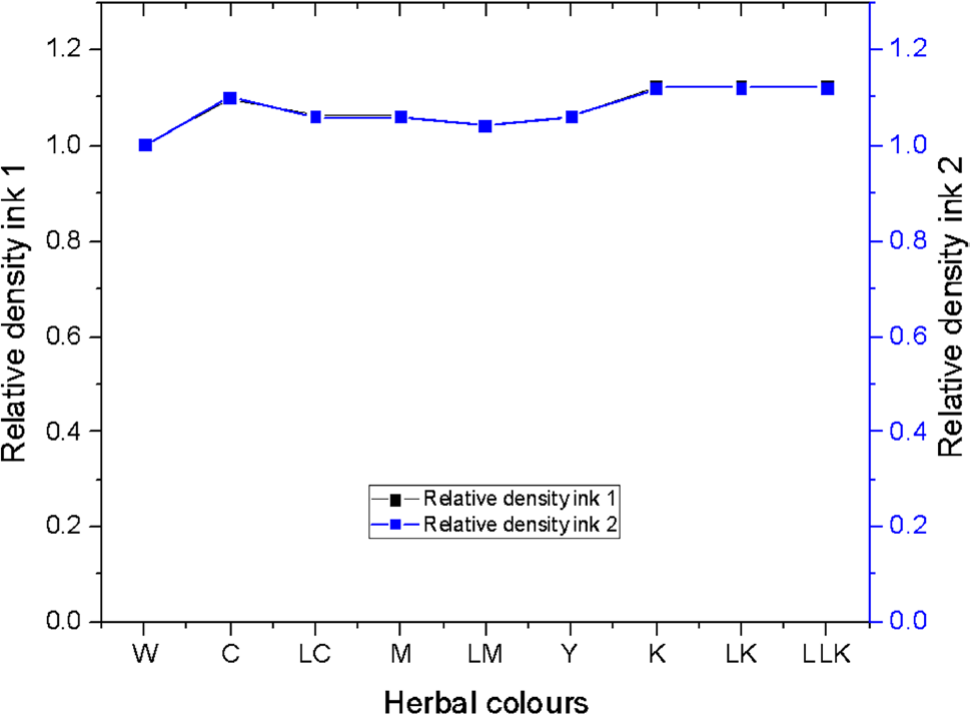
Relative density graph for plant-based inks 1 and 2. Note: The legend given within Fig. 8 applies to all the graphs, figures, and tables that are organised henceforth, comprising W, C, LC, M, LM, Y, K, LK, and LLK.

The relative density as acquired for PI1 and PI2 is plotted in Fig. 9, showing similar values under the current study. The constituted black colour (K, LK, and LLK) plant-based inks had a maximum relative density of 1.12. The plant-based ink formulated from quebracho plant extract for magenta colour (M) acquired the minimum relative density of 1.04. The relative density differed for each colour; the discrepancy could be attributed to the particle size of the plant extracts. Finally, the ANOVA one-way test results concluded the relative density value of 1.06 to be the most influential on the K/S values gained on the wool and cotton fabrics inkjet printed with plant-based inks.

#### Viscosity

**Fig. 10:**
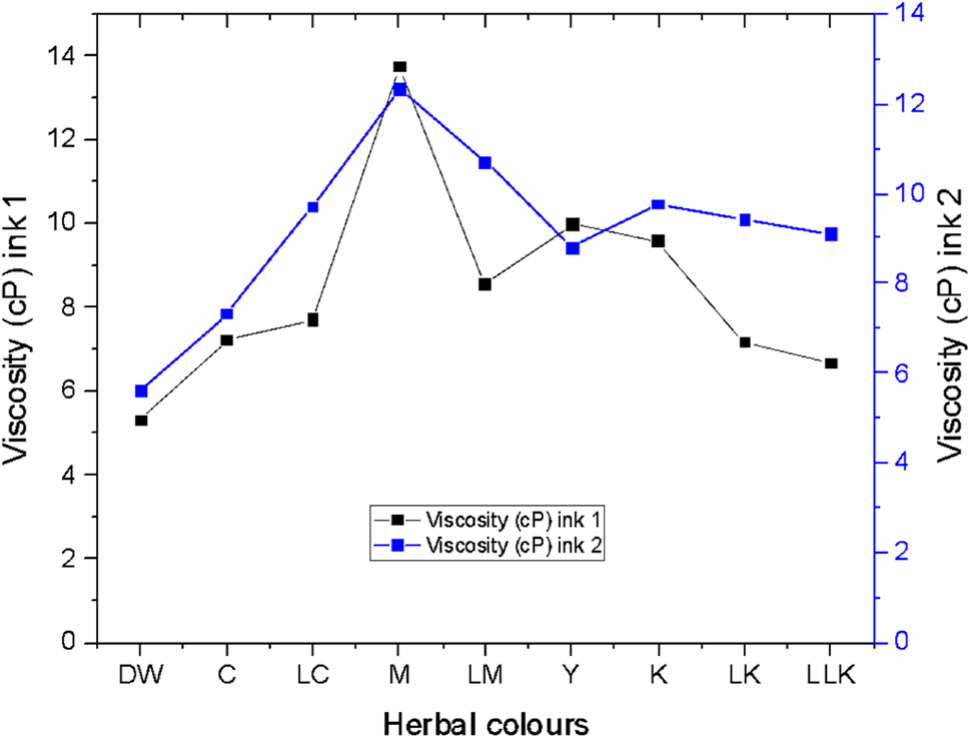
Viscosity graph for plant-based inks 1 and 2.

The viscosity measured at 24 °C and 180 rounds per minute (RPM) is graphed in Fig. 10, where the high-powered rheometer yielded zero-shear viscosity and likewise zero-shear rate viscosity for all the two sets of plant-based inks. It indicates extremely low shear conditions. Likewise, it denotes plant-based inks to be Newtonian in nature. The zero-shear viscosity is considered one of the valuable rheological metrics for analysing Newtonian ink behaviour in the storage phase (Hodges, 2021) (Patton, 1979). In this study, PI1 obtained the highest viscosity value of 13.73 cP with the ink made from the quebracho plant extract for magenta colour (M). The second highest viscosity value of 9.98 cP was noted with the ink made from the logwood plant for yellow colour (Y), followed by the viscosity value of 9.56 cP obtained with the plant-based ink formulated for the black colour (K). Similarly, PI2 demonstrated the highest viscosity value of 12.30 cP with ink constituted from the quebracho plant for magenta colour (M). The second highest viscosity value of 10.70 cP was noted for the ink formulated from the quebracho plant for light magenta colour (LM), followed by the viscosity value of 9.76 cP gained with the plant-based ink formulated for the black colour (K). Practically, a similar trend of viscosity was noted for PI1 and PI2. Ultimately, the ANOVA one-way test results determined the viscosity value of 9.98 cP to be the most influential on the K/S values gained on the wool and cotton fabrics inkjet printed with plant-based inks.

#### Surface tension

**Fig. 11:**
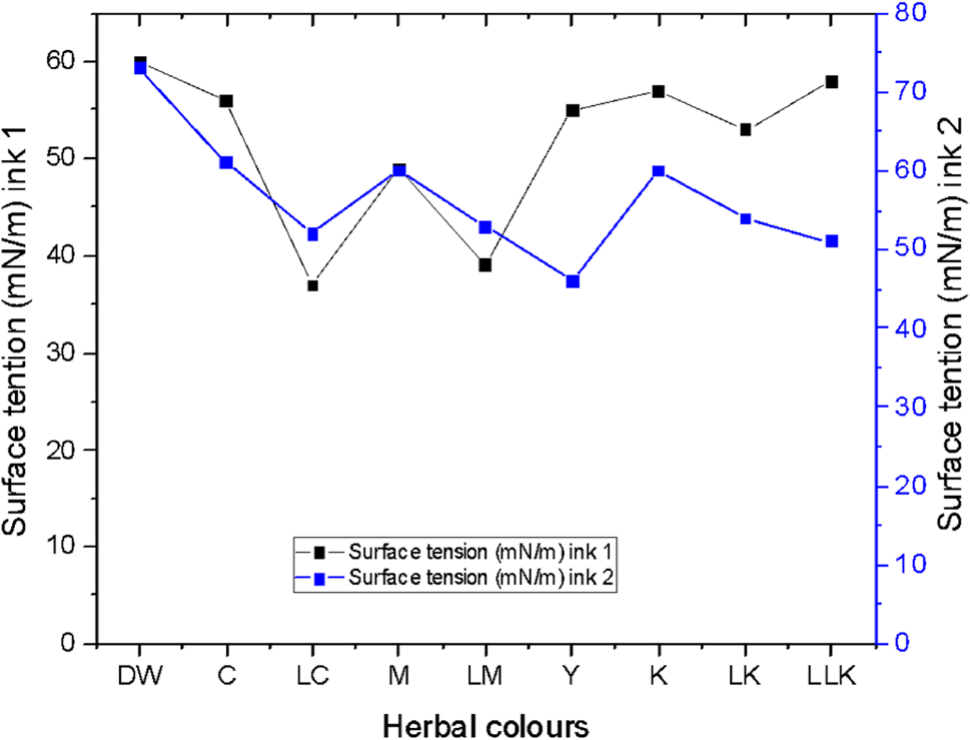
Surface tension graph for plant-based inks 1 and 2.

Each colour in the developed palette manifested distinct characteristics as its ink droplet interacted with the cotton fabric surface. In Ink 1, the droplet spheres for most colours exhibited a faster propulsion compared to those in Ink 2. However, exceptions were observed for LC and LM, where the spread took a longer time to cover a larger area. The surface tension of both developed inks is visually depicted in Fig. 11. Ink 1 demonstrated the maximum surface tension value of 58 mN/m for light, light black colour (LLK). The LLK in ink 1 is formulated with the plant-based mixture of bio indigo leaf extract blue colour (C), quebracho plant red colour (M), and logwood plant yellow colour (Y). The smallest surface tension value of 37 mN/m was noted for cyan colour (C) plant-based ink constituted from the bio indigo leaf extract. Conversely, for ink 2, the greatest surface tension of 61 mN/m, was indicated for cyan colour (C) plant-based ink formed from bio indigo leaf extracts. The least surface tension value of 46 mN/m was noted for the yellow colour (Y) plant-based ink devised from the flame of the forest flowers extract. After all, the ANOVA one-way test results concluded the surface tension value of 60 mN/m to be the most influential on the K/S values gained on the wool and cotton fabrics inkjet printed with plant-based inks.

Considering viscosity and surface tension together of the new inks from plants, convincingly, as revealed in the literature, the viscosity value of less than 10 cP propels appropriate spherical drop formation. A higher ink viscosity value would lower the speed of the ink drop producing an ink drop with a long-tail length (Sun, et al., 2012a). Likewise, the surface tension above 35 mN/m would facilitate a high speed of ink drop as required for the inkjet print technology. The higher surface tension would further impel sphere ink drop with a short tail length (Sun, et al., 2012b). The result derived with rheological parameters is organised in Table 12. Altogether, the obtained empirical findings broadly support the work of previous studies in this area, linking viscosity and surface tension to the quality of the colours and prints acquired on inkjet printing.

#### Conductivity

**Fig. 12:**
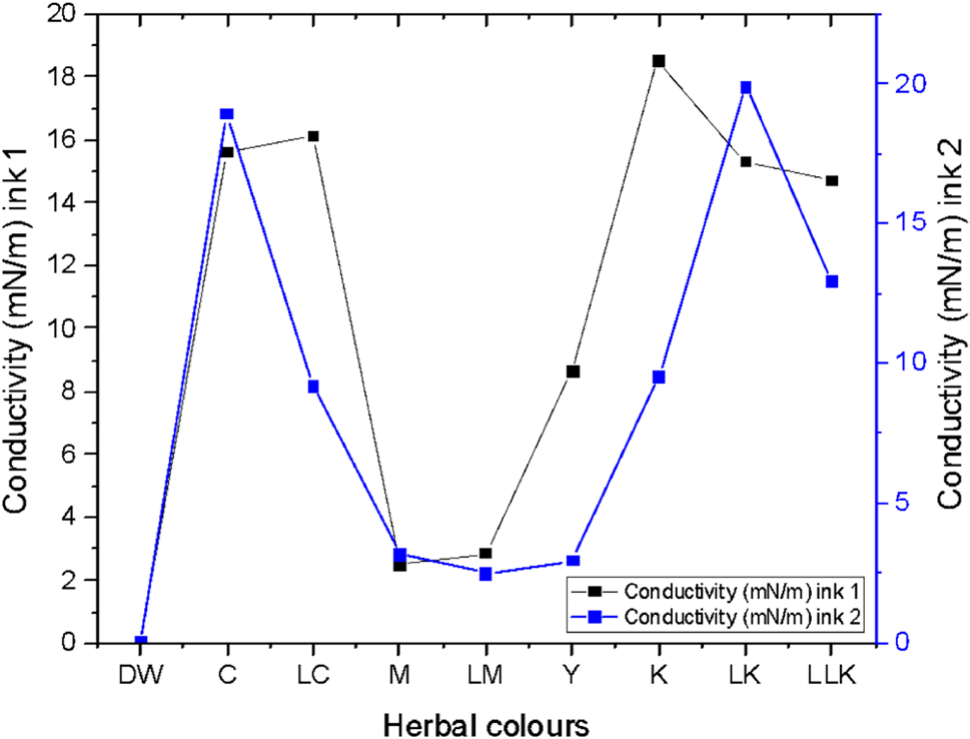
Conductivity graph for plant-based inks 1 and 2.

The conductivity graph for inks 1 and 2 is demonstrated in Fig. 12. Ink 1 denoted a maximum conductivity measure of 18.5 mS/cm for black colour (K) plant-based ink and the minimum conductivity measure of 2.51 mS/cm for quebracho red bark extract ink for magenta colour (M). Similarly, ink 2 indicated a maximum conductivity measure of 19.91 mS/cm for light black colour plant-based ink (LK) and a minimum conductivity measure of 2.51 mS/cm for the ink prepared from quebracho red bark for light magenta colour (LM). The graph clearly illustrates a similar trend of increase and decrease of conductivity values for PI1 and PI2. Lastly, the ANOVA one-way test results concluded the conductivity value of 2.51 mS/cm to be the most effective on the K/S values gained on the wool and cotton fabrics inkjet printed with plant-based inks. The high conductivity values could be of vital importance while the digital printing of microelectronic surfaces. Moreover, the plant-based inks could be combined with graphene or silver or gold for acquiring higher conducting inks ecologically.

#### pH

**Fig. 13:**
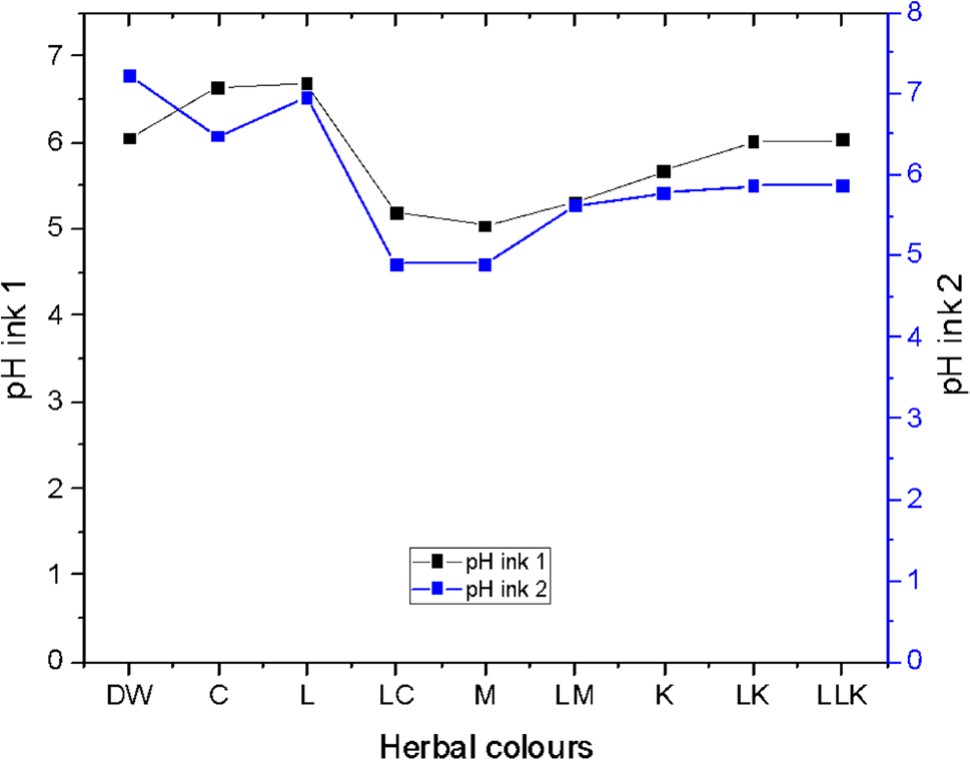
pH graph for plant-based inks 1 and 2.

The pH values of PI1 and PI2 are demonstrated in Fig. 13. Ink 1 denoted the acidic pH value of 5.03 for the quebracho plant ink for magenta colour (M), and a nearly neutral pH value of 6.68 was observed for bio indigo leaf extract ink for light cyan colour (LC). Likewise, ink 2 indicated an acidic pH value of 4.90 for both quebracho red bark extract inks for magenta and light magenta colours (M and LM). Also, a nearly neutral pH value was studied for plant-based ink prepared from the bio indigo leaf extract for light cyan colour (LC). Again, a similar trend of pH values was observed for PI1 and PI2. Finally, the ANOVA one-way test results concluded the pH value of 4.9 to be the most influential on the K/S values gained on the wool and cotton fabrics inkjet printed with plant-based inks.

This research is the only comprehensive investigation conducted on water-based inks constituted from plant extracts for digital printing of textile substrates. The colours gained were blue, red, yellow, and black instead of cyan, magenta, yellow, and black, colours correspondingly still promising. The challenging black colour was derived from the mixture of primary plant-based colours, which is encouraging. The research findings on stoichiometry and rheology contribute in several ways to understanding plant-based inks and provide the basis for their effect on colour values, inkjet printing, and storage stability.

The rheological property of formulated plant-based inks was anticipated to differ with each packed plant procured from varied sources. Hence, the analysis was of vital importance. However, graphically, no drastic fluctuations were noted in-between the formulated inks 1 and 2. Overall, as mentioned in the literature review, the physical parameters of plant-based inks were acceptable for both plant-based inks 1 and 2. Additionally, it reinforces the reliability of the adapted stoichiometry of plant-based inks. Following the stoichiometry (chemistry) accurately would yield plant-based inks with appropriate rheological (physical) properties.

### Stability of plant-based inks

The developed inks were assayed for stability to a one-month storage phase in the shade at room temperature. The freshly prepared plant-based ink is denoted as NPI/N. The same ink was stored for one month, indicated as OPI/O for one-month-old plant-based Ink. The OPI and NPI were examined with 3 approaches: (1) visual examination, (2) assessment using ATR-FTIR, and (3) rheological property study as discussed herein for C, M, Y, and K plant-based colours.

**Fig. 14:**
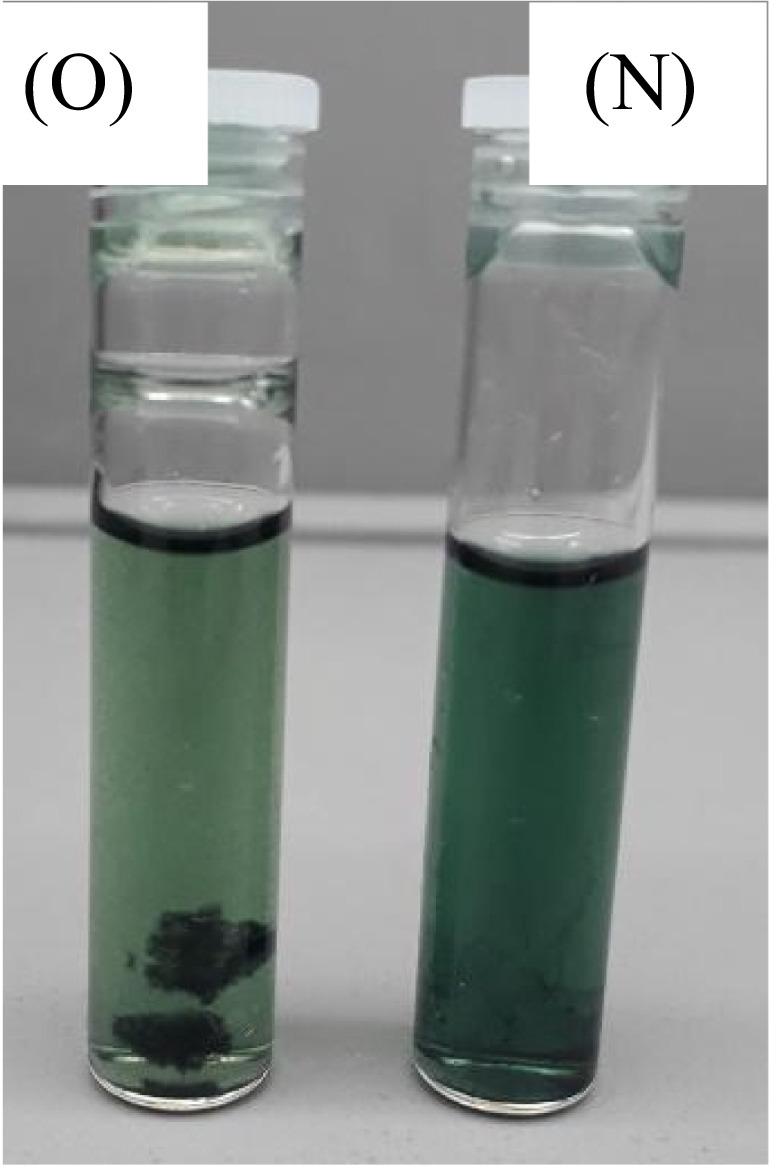
Cyan (C) colour OPI and NPI under D65.

1. Visual examination was conducted in the colour inspection lightbox under D65 illumination. The Verivide CAC 150 colour assessment lightbox was applied for evaluating the OPI and NPI, as shown in Figures 14, 16, 18, and 20. Each of the plant-based ink was bottled in a sterile air-tight clear container when freshly prepared. It was placed in the lightbox for visual examination of plant-based inks

(2) ATR-FTIR analysis was performed on Thermo Scientific™, Nicolet™ iS™ 5 ATR-FTIR Spectrometer with iD7 ATR–diamond accessory. The ATR-FTIR investigation would study the change in peak functional groups, also known as phytochemicals, in plant-based inks due to the storage phase, as indicated in Figures 14, 16, 18, and 20. The readings were noted at percentage (%) reflectance mode under controlled experiential parameters

(3) Rheological assessment of plant-based inks was performed for C, M, Y, and K colours. The physical parameters, namely, viscosity, surface tension, conductivity, and pH of one-month OPI, were contrasted with freshly prepared NPI of C, M, Y, and K colours, as presented in Figures 21, 22, 23, and 24

#### Bio indigo leaf extracted blue (C) colour inks

##### Visual examination

As illustrated in Fig. 14, the OPI from bio indigo leaf extract for C colour demonstrates sediments during a 1-month storage phase. A possible explanation for this result may be the air passage into the container. It bears a resemblance to algal growth. The NPI colour is observed to be deeper in contrast to OPI prepared from bio indigo leaf extract. This difference could be explained as an impact of lot-to-lot variation in the source of bio indigo leaf extract products and or change in phytochemistry of plant-based ink due to storage.

##### ATR-FTIR analysis

**Fig. 15:**
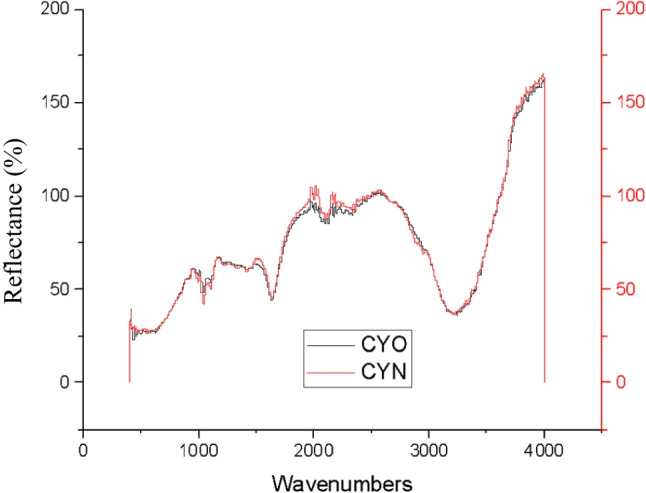
ATR-FTIR analysis of cyan (CY) colour OPI and NPI.

As revealed in Fig. 15, the OPI and NPI of C colour demonstrated identical peak, functional groups, at the wavenumbers of 1040.15 cm^−1^ for strong C-O alkoxy band, 1415.38 cm^−1^ for moderate C-C stretch (in-ring) of aromatics, and at 1633.50 cm^−1^ wavenumbers for moderate N-H band of 1° amine were indicated. At 2122.80 cm^−1^, wavenumbers weak C≡C alkynes stretch, and strong 3227.23 cm^−1^ of O-H stretch and H-bonded for alcohols and phenols groups were observed, indicating decent chemical stability to one-month storage conditions.

#### Quebracho red bark extracted red (M) colour inks

##### Visual examination

**Fig. 16:**
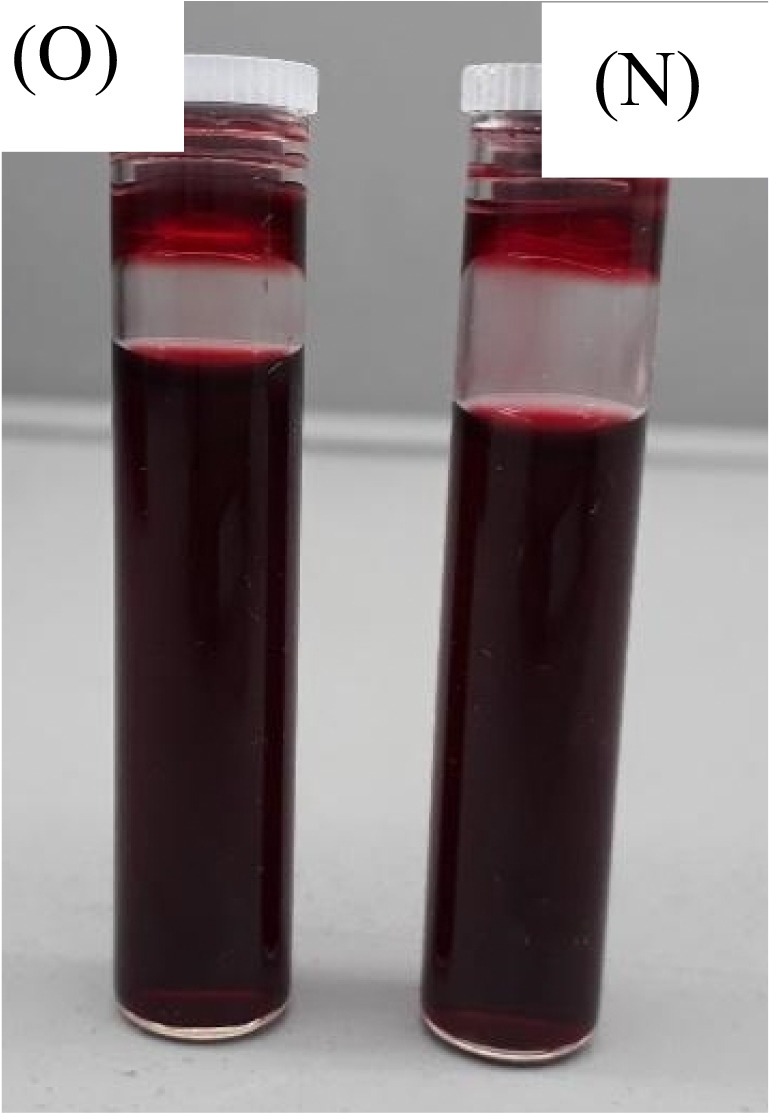
Magenta (M) colour plant-based inks OPI and NPI under D65.

The old and new inks for the M colour prepared from the quebracho red bark extract exhibited no obvious colour change in the storage phase after one month, as presented in Fig. 16.

##### ATR-FTIR analysis

**Fig. 17:**
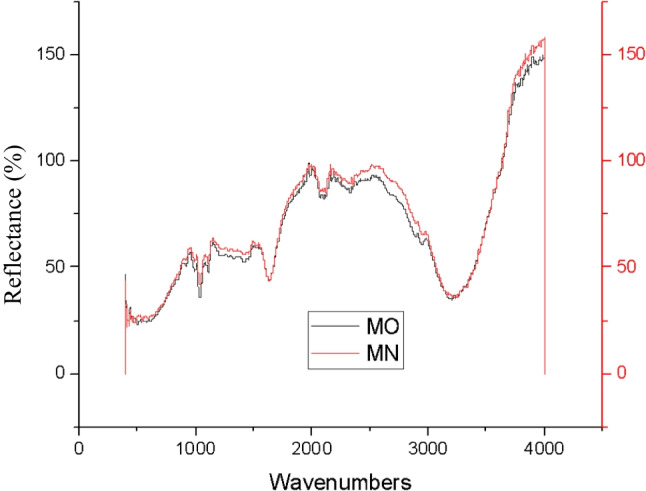
ATR-FTIR analysis of magenta (M) colour plant-based inks OPI and NPI.

As demonstrated in Fig. 17, the OPI and NPI of M colour exhibited similar peak, functional groups, at the wavenumbers of 1037.77 cm^−1^ and 1110.03 cm^−1^ strong C-O stretches of alcohols, carboxylic acids, esters, and ethers. At wavenumbers of 1414.34 cm^−1^ for moderate C-C stretch (in-ring) of aromatics were noted. Additionally, at wavenumbers, 1632.48 cm^−1^ moderate N-H bends of 1° amines, at 2100.34 cm^−1^ and 2331.82 cm^−1^ weak C≡C of alkynes, and 3208.23 cm^−1^ wavenumbers moderate O-H stretch of carboxylic acids were observed, hence, denoting adequate chemical stability to the 1-month storage phase.

#### The flame of the forest flowers extracted yellow (Y) colour inks

##### Visual examination

**Fig. 18:**
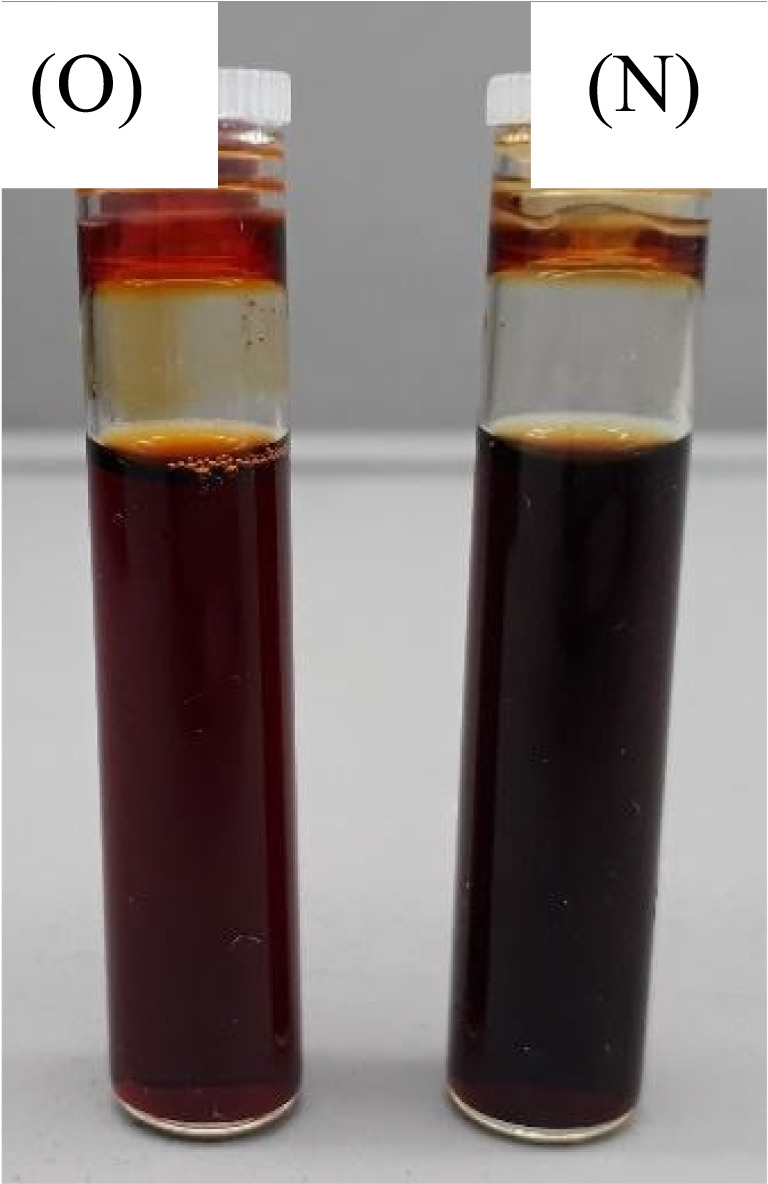
Yellow (Y) colour plant-based inks OPI and NPI under D65.

The old and new yellow colour (Y) plant-based ink constituted from the flame of the forest flowers extract was found to be identical, as shown in Fig. 18.

##### ATR-FTIR analysis

**Fig. 19:**
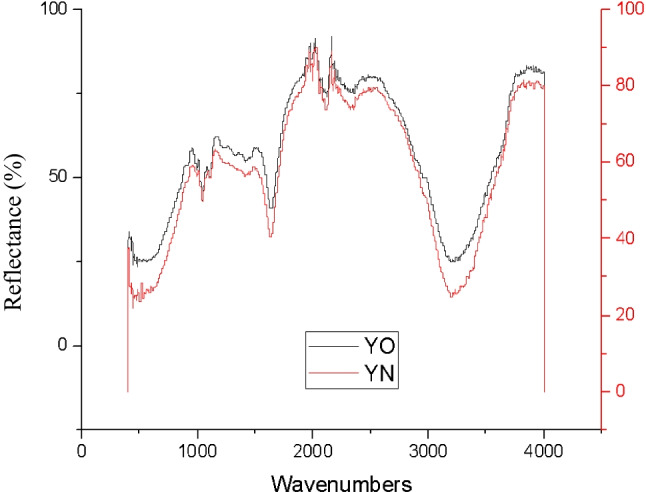
ATR-FTIR analysis of yellow (Y) colour plant-based inks OPI and NPI.

The OPI and NPI of Y colour depicted similar peak functional groups at wavenumber 1040.38cm^−1^ strong C-O stretch of alcohols, carboxylic acids, esters, and ethers were indicated. At wavenumbers, 1633.34 cm^−1^ moderate N-H bends of 1° amine were noted, and at 3225.26 cm^−1^ wavenumbers, moderate O-H stretch was demonstrated. The additional peak functional groups were observed at 992.45 cm^−1^ strong =C-H bend of alkenes and at 1109.47 cm^−1^ of wavenumbers moderate C-N stretch of aliphatic amines was illustrated for the OPI of Y colour as illuminated in Fig. 19. The reason for this inconsistency between OPI and NPI is not apparent; hence, detailed analysis would be essential for further predictions with enhanced accuracy. The inconsistency may be due to source errors associated with plant materials.

#### Black colour (K) plant-based inks

##### Visual examination

**Fig. 20:**
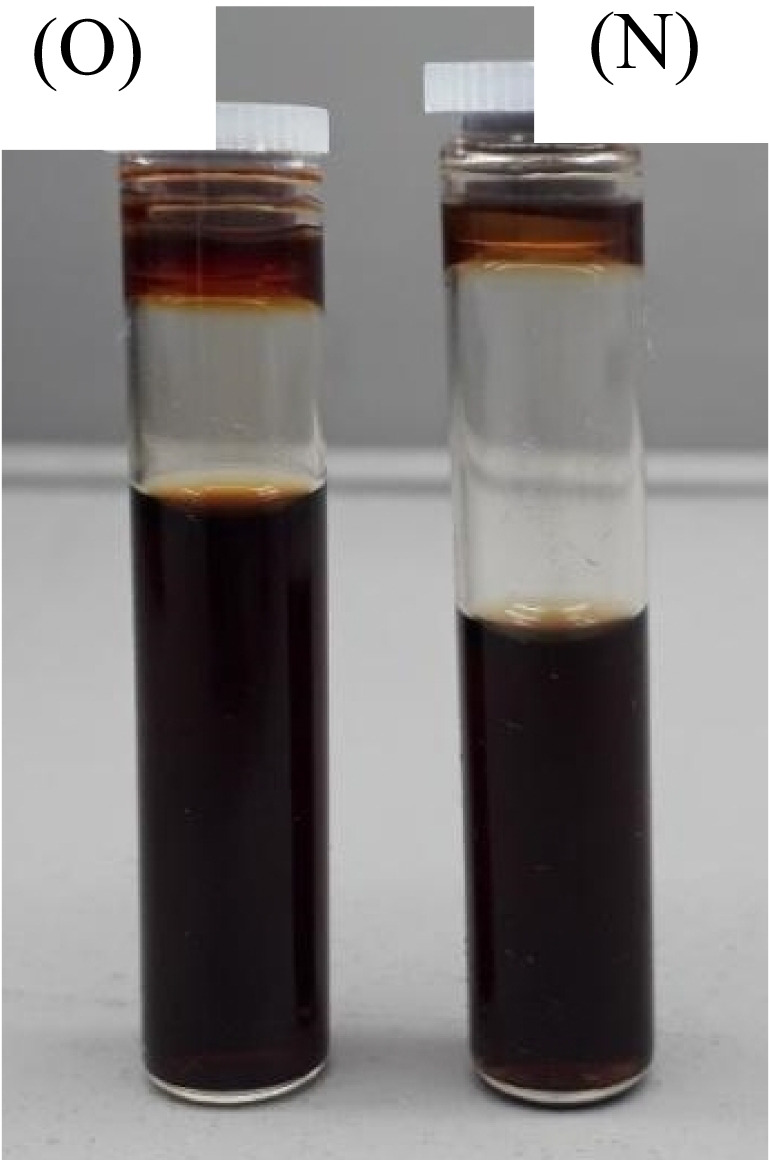
Black (K) colour plant-based inks OPI and NPI under D65.

The old and new black (K) colour plant-based inks demonstrated minute residues of plant extracts settled at the bottom of the bottled plant-based ink solutions, as displayed in Fig. 20.

##### ATR-FTIR analysis

**Fig. 21:**
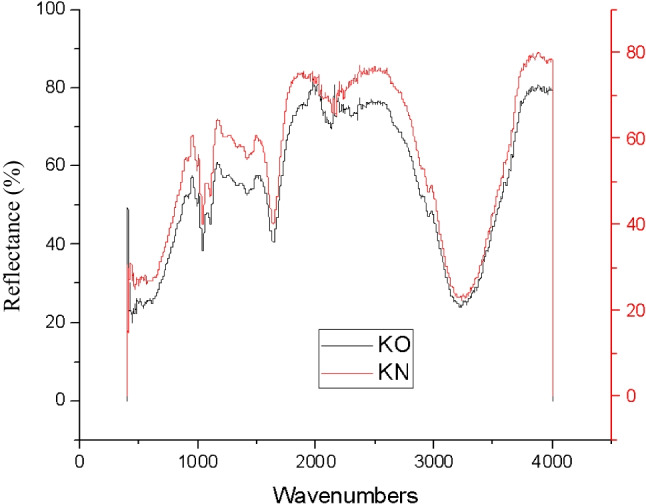
ATR-FTIR analysis of black (K) colour plant-based inks OPI and NPI.

The peak phytochemical units were indicated at the wavenumbers 464.82 cm^−1^, 1039.68 cm^−1^, and 1106.97 cm^−1^, and a strong C-O stretch of alcohols, carboxylic acids, esters, and ethers was illustrated. At 1640.40 cm^−1^ moderate -C=C- a stretch of alkenes and 3234.24 cm^−1^ wavenumbers, a moderate N-H stretch of 1°, 2° amines, and amides were indicated for NPI of K colour. The OPI of K colour denoted additional peak, functional groups, at the wavenumbers 536.01 cm^−1^ and 992.55 cm^−1^, strong =C-H bend of alkenes was noted as demonstrated in Fig. 21.

As mentioned above, the old plant-based ink (OPI) was assessed after one month of the storage phase. The new plant-based ink (NPI) was evaluated when freshly prepared. Taken together, one of the issues that emerge from these findings is the sediments occurring in the bio indigo leaf extract. Nitrogen blanketing is suggested to prevent the ingress of air, thereby eliminating the risk of atmospheric contamination of plant-based inks. Similarly, nitrogen purging is recommended wherein the inert high purity compressed N_2_ gas that 99.99% (Linde plc., 2013) would purge the oxygen from the glass bottles before sealing hence preserving and protecting the plant-based ink product from oxidation, due to oxygen content from the air and or hydrolysis due to moisture content from the air (MVS Engineering Pvt. Ltd., 2021). The strong triple bond in-between two molecules of nitrogen that is N≡N is covalent in nature. It requires high energy to disassociate this molecule and take part in any other chemical reaction. Hence, the N_2_ gas remains chemically non-reactive under normal conditions (Vedantu.com, 2011).

Additionally, a closer inspection reveals that the K, LK, and LLK coloured inks constituted from the mixture of bio indigo leaf extract, quebracho plant, and the flame of the forest flowers extract in the proportion of 9:1:1 individually exhibit no sediments as observed for bio indigo leaf extract inks of C and LC colours. This rather contradictory result may be due to the higher photochemical stability of the quebracho plant, and the flame of the forest flower extract incorporated in the formulation of K colour plant-based ink. The quebracho plant and the flame of the forest flowers extract intrinsically enclose high tannin and butein content, respectively, stated to be a functionally efficient antioxidant and antibacterial (Dave, Darji, & Gandhi, 2019) (Panzella, et al., 2019); hence, no alien growth or sediments were observed. Moreover, a deeper probe is recommended to accurately determine the change in peak functional groups of phytochemicals in OPI and NPI. According to the literature review, the ATR-FTIR analysis could be corroborated with Raman spectroscopy and optical absorption spectroscopy and atomic force microscopy for the characterisation of inks according to literature review (Carey, 2017).

### Rheological properties of inks in the storage phase

**Fig. 22:**
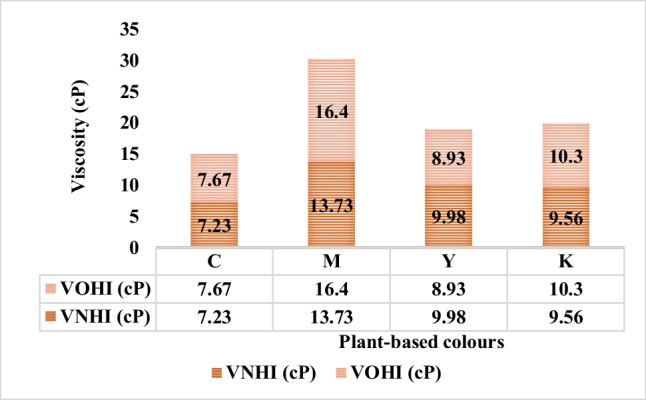
The viscosity values of old and new C, M, Y, and K colour plant-based inks. Note: The H is for plant-based inks.

**Fig. 23:**
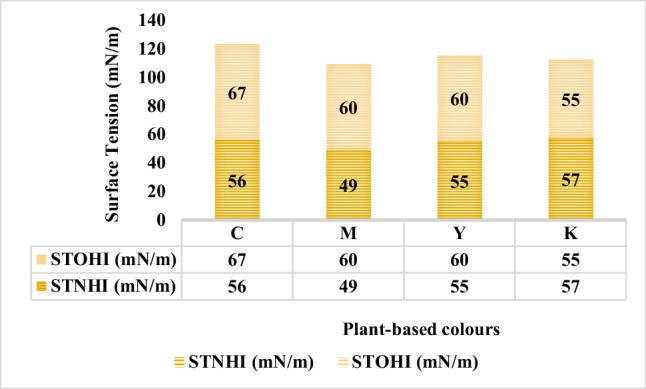
The surface tension values of old and new C, M, Y, and K colour plant-based inks.

**Fig. 24:**
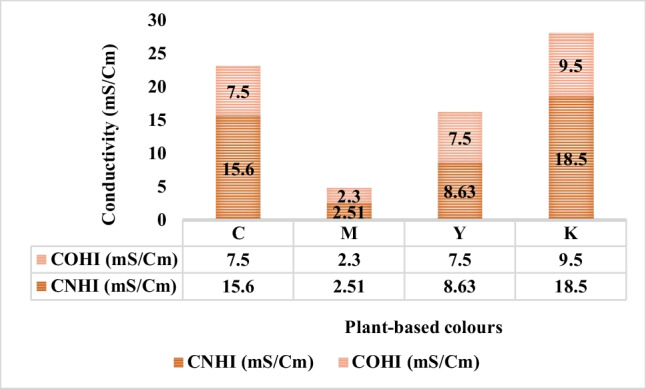
The conductivity values of old and new C, M, Y, and K colour plant-based inks.

**Fig. 25:**
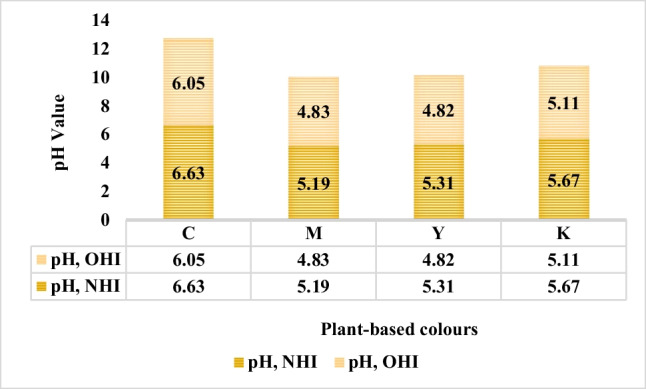
The pH values of old and new C, M, Y, and K colour plant-based inks.

The C, M, Y, and K coloured inks were examined to change physical properties concerning viscosity, surface tension, conductivity, and pH parameters, as presented in Figures 22, 23, 24, and 25, respectively. The rheology of freshly prepared inks was contrasted with old inks after the 1-month storage phase. The rule of thumb indicates that the smaller viscosity values likewise, shorter surface tension and lesser conductivity values, are desirable for drop-on-demand inkjet print technology. The range of each is elaborated in the literature review.

The change in viscosity of inks in the storage phase after 1 month was determined on the Brookfield DV-II + Pro Viscometer. Figure 22 graphically depicts the viscosity values for OPI and NPI for cyan (C), magenta (M), yellow (Y), and black (K) colours. Surprisingly, the old inks exhibited a higher viscosity value in contrast to the new inks, except for the yellow-coloured ink. Most distinctly, the viscosity value of 16.4 cP was observed for 1-month-old magenta-coloured ink in contrast to the viscosity value of 13.73 cP studied for the freshly prepared magenta-coloured ink.

The viscosity could decrease with time, as demonstrated by Y-coloured ink, and return to its original value after a period of rest; such behaviour is known as thixotropic. Conversely, the increase in viscosity during a period of rest is termed anti-thixotropy aka rheopexy (Patton, 1979). Hence, anti-thixotropy was reflected by C, M, and K coloured inks. Altogether since the viscosity no longer remains constant, the lant-inks are designated non-Newtonian. The commercial links are rarely Newtonian in nature (Patton, 1979).

The change in surface tension of old and new inks was tested on KRUSS manual force tensiometer—K6. Figure 23 illustrates the change in surface tension values in-between old and new plant-based inks for C, M, Y, and K colours.

The single most striking feature to emerge from the graphical data is that the surface tension increases for the C, M, and Y colour inks in the storage phase after one month, whereas the new C, M, and Y inks demonstrate lesser surface tension. The K-coloured ink is an exception wherein the surface tension value of the one-month-old black coloured ink is 55 mN/m, and the surface tension value of freshly prepared black ink is 57 mN/m. One of the plausible explanations to justify the change in the surface tension of plant-based inks could be due to a probable change in the stoichiometry of the dissolved solids in plant-based inks due to the storage phase. The reasons providing for surface tension oscillations include Van der Waals’ forces of attraction among polar groups of the formulated solutions (plant-based inks) as postulated by Dutch physicist Johannes Diderik van der Waals in 1873. Also, the universal dispersion forces among polar groups of the constituted colloidal mixtures (plant-based inks) as recognised in 1930 by the Polish-born physicist Fritz London hence aka London forces. Additionally, hydrogen bonding contributes to the surface tension fluctuations. Herein, the higher the bond formations the greater would be the surface tension values and vice versa (Shaw, 1980) (Encyclopædia Britannica, Inc., 2022b).

The conductivity was evaluated on Hanna instrument conductivity metre HI 8733. Figure 24 depicts the change in conductivity values of C, M, Y, and K inks after one month of the storage phase.

It is evident from Fig. 24 that the conductivity values for the new C, M, Y, and K inks are higher as compared to their old counterparts. The conductivity of the solution changes with the concentration changes of dissolved ions in the solution, changes in the types of ions, and the temperature of the solution (Apera Instruments, LLC, 2018). It seems possible that these results are due to indicated changes in the 1-month storage phase of inks. The most drastic difference in the conductivity value was noted for OPI and NPI of K colour, the values being 9.5 and 18.5, respectively.

The pH metre assayed the change in pH of the old and new C, M, Y, and K inks. Hanna HI 8424 pH/mV/°C was implemented for the pH assessment of plant-based inks formulated from the plant extracts.

As illustrated in Fig. 25, the pH values of the new C, M, Y, and K inks were marginally higher than their old counterparts. The pH of inks remained acidic after the 1-month storage phase. For example, the cyan ink formulated from the bio indigo leaf extracts blue plant extract indicated a pH value of 6.63 when freshly prepared. However, the pH dropped to 6.05 after the 1 month. A drop in pH in the storage phase with a subsequent increase in viscosity parameter is frequently noted with commercial inks (Podhajny, 2004). A similar trend was observed for other inks. Furthermore, it causes the ink to foam, the ink dry-up prematurely, and deteriorates the print quality producing ragged edges on printing (Podhajny, 2004). It accurately justifies the problem faced with DOD printing on wool and cotton fabrics with the developed inks. It is anticipated that neutral pH could dissipate the problems associated with colloidal ink solution, however, uncertain (Podhajny, 2004). In contrast, for natural colours in specific, it is pragmatic (the rule of the thumb) that for proteinic fabric colouration, acidic pH is favourable, whereas for cellulosic fabric colouration, alkaline pH is favourable (Cardon, 2007) (Fereday, 2003). In general, since the change in rheology, viscosity, surface tension, conductivity, and pH, over the 1-month storage phase remains in range as detailed in the literature review, and the inks would continue to print. For synthetic inks, it is recommended to be utilised within 3 months of opening, while the packed ink has a shelf-life of more than a year. Hence, taken into consideration together, these findings have important implications for developing plant-based inks with longer stability to storage time.

Overall, this study strengthens the idea that plant-based biomaterials are incredibly prospective for formulating plant-based inks, plant-based pre-treatments, and plant-based washing off recipes. According to ANOVA one-way test analysis, the concluding parametric study with plasma surface-treated wool and cotton fabrics digitally printed with the plant-based inks achieved a *p* value of 0.0004630 and an actual power of 0.99, which is reassuring. To end, the plant-based inks with viscosity value between 8.5 and 10 cP, relative density of 1.06, surface tension of 60 mN/m, conductivity of 2.51 mS/cm, and a pH of 4.8 were inferred to be appropriate for inkjet printing of wool and cotton fabrics. These are the beneficial theoretical implications of the present study. In essence, the waterless technology approach will prove helpful in expanding the colour range accessible with natural colours. At the same time, the amount of extract is concentrated in plant-based ink formulation; hence, significantly less quantity is consumed on inkjet printing than for dyeing. Consequently, it would defend agricultural land from being jeopardised for cultivating plants for fabric dyeing. The practical implication of this experiential research is that it would save both water and plants, as a result, propel climate action. Before this study, evidence of digital printing with plant-based formulations was purely anecdotal.

### Statistical analysis

The factorial experiment designed for the study was validated with the ANOVA one-way test on Origin Lab software 2018b, 64bit for analysis. Stratified random sampling was applied in the study to assert that each segment in a population is similarly represented (Shin, 2020). The ANOVA one-way test parameters are organised in Table 9.

**Table 9:** ANOVA one-way test parameters were applied for the study.

ANOVA one-way parameters	Category
Input data	Indexed
Statistics	Descriptive
Significance level	0.05
Tests for equal variance	Levene l l
Power analysis	Actual power
Plots	Tukey plot/Bonferroni plot and means plot SE and box plot

*Viscosity variance and K/S response*

To identify the effect of the viscosity variance on the K/S data response, the ANOVA one-way test was conducted. The data implemented for the study is organised in Table 10.

**Table 10:** Data utilised for analysis of viscosity variance and K/S response.

FN	PC	Viscosity variance	Viscosity range	K/S response data
A1	Bio indigo leaf extract blue	7.7	L	3.2294
A2	Logwood plant yellow	9.98	M	3.9606
A3	Quebracho plant red	13.73	H	3.8668
A4	Bio indigo leaf extract blue	7.7	L	3.126
A5	Logwood plant yellow	9.98	M	3.6058
A6	Bio indigo leaf extract blue	7.7	L	0.8158
A7	Quebracho plant red	13.73	H	2.1428
A8	Purple (black colour)	9.56	M	3.4913
B1	Bio indigo leaf extract blue	7.7	L	3.6396
B2	Purple (black colour)	9.56	M	0.8509
B3	Purple (black colour)	9.56	M	0.897
B4	Quebracho plant red	13.73	H	5.5938
B5	Quebracho plant red	13.73	H	3.0909
B6	Bio indigo leaf extract blue	7.7	L	0.9337
B7	Pink (black colour)	9.56	M	3.0843
B8	Bio indigo leaf extract blue	7.7	L	3.167

**Fig. 26:**
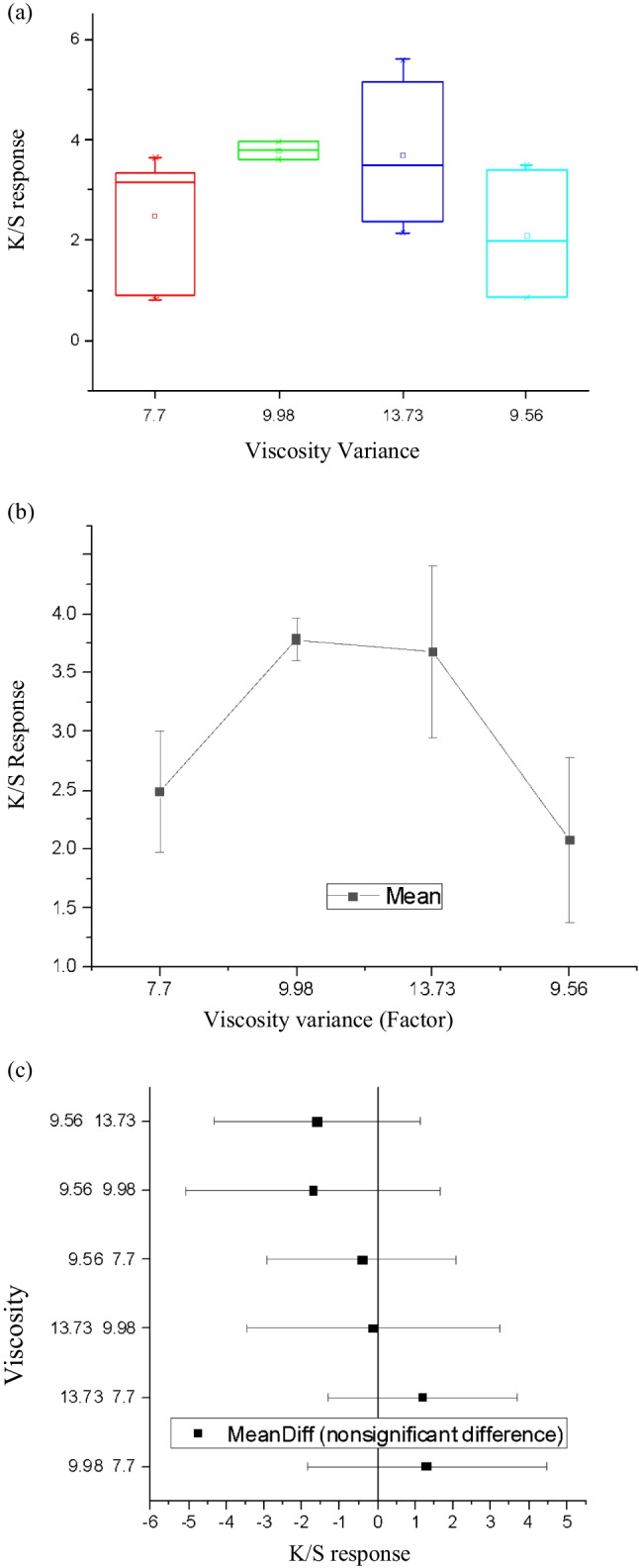
Charts of ANOVA one-way test results for plant-based inks parameter **a** box plot, **b** standard error of the mean plot, and **c** Tukey graph.

Referring to charts in Fig. 26a–c and the statistical data displayed in Table 11, the most reliable data corresponding to viscosity is 9.98 cP, which corresponds to the K/S value of 3.9606. Therefore, as per the viscosity scale adopted, the viscosity range of below 10 cP and above 8.5 cP was agreed to be the best for further parametric study. It corresponds to the medium-level viscosity range, illustrated in Table 8. However, a probability (>*F* value) was 0.26497, and the Tukey test denoted Sig 0, indicating that the difference of the means is not significant at the 0.05 level.

**Table 11:** Statistics gained on viscosity variance and K/S response.

Viscosity range	Mean	SD	SE
7.7	2.48	1.26	0.51
9.98	3.78	0.25	0.17
13.73	3.67	1.46	0.73
9.56	2.08	1.40	0.70

Convincingly, as revealed in the literature, the viscosity value of less than 10 cP propels appropriate spherical drop formation. A higher ink viscosity value would lower the speed of the ink drop producing ink drop with long-tail length (Sun, Wei, & Huang, Influence of the Viscosity of Edible Ink to Piezoelectric Ink-jet Printing Drop State, 2012). Likewise, the surface tension above 35 mN/m would facilitate a high speed of ink drop as required for the inkjet print technology. The higher surface tension would further impel sphere ink drop with a short tail length (Sun, Wei, & Huang, Influence of the Surface Tension of Edible Ink to Piezoelectric Ink-jet Printing Drop State, 2012). The result derived with rheological parameters is organised in Tables 11 and 12. Altogether, the obtained empirical findings broadly support the work of previous studies in this area, linking viscosity and surface tension to the quality of the colours and prints acquired on inkjet printed fabric substrate.

**Table 12:** Statistics gained on plant-based inks rheology.

Rheology	Most effective value	SD	SE	*P* > *F*	Sig value
Relative density	1.06	1.19	0.48	0.41	0
Viscosity	9.98	0.25	0.17	0.26	0
Surface tension	60	0.59	0.34	0.32	0
Conductivity	2.51	0.55	0.39	0.29	0
pH	4.9	0.67	0.47	0.36	0

## Conclusions and implications

This research is the only comprehensive investigation conducted on water-based inks constituted from the plant extracts for digital printing of textile substrates. The conclusions are as follows.

1. The plant-based inks with viscosity value between 8.5 and 10 cP, relative density of 1.06, the surface tension of 60 mN/m, the conductivity of 2.51 mS/cm, and pH of 4.8 were inferred to be appropriate for inkjet printing of wool and cotton fabrics. These are the empirical and theoretical implications of the present study
2. Overall, the physical properties of plant-based inks were stable for one-month storage time at room temperature
3. The water-based eco-friendly inks illustrated no sediments or precipitation over the storage period except for blue-coloured ink constituted from the bio indigo plant extract for which the nitrogen blanketing of the storage bottles is recommended to prevent the ink spoilage due to ingress of atmospheric air and water
4. As hypothesised for the study, the probability values of 0.41, 0.26, 0.32, 0.29, and 0.36 were observed for the relative density, viscosity, surface tension, conductivity, and pH correspondingly and the Tukey test denoted Sig 0 for each parameter, indicating that the difference of the means is not significant at the 0.05 level

The comprehensive assessment of the properties of the plant-based inks for inkjet printing on textiles provided promising results. It implies the potential to revolutionise inkjet printing for the textile industry by utilising eco-friendly alternatives for sustainable practises. The research outcome aligns with the need for the global shift towards greener technologies.

## Future work

The constituted plant-based inks are inherently dense in bioactive plant phytochemicals that impart functional properties, namely, anti-microbial, anti-fungal, and others, to the natural textile substrate. This aspect could be explored in the forthcoming research study. Bio indigo leaf extract ink required to be stabilised for a longer shelf-life. Billions of colours could be generated from the plant-based basic blue, red, yellow, and black colour inks on digital printing. It is the potential future research and development area with plant-based inks formulations. The planted-based inks require to be investigated for the print quality acquired on the inkjet printed fabrics, coffee-ring effect, and the plausible phytochemistry of inks. Additionally, the research findings on the intrinsic conductive property of plant-based inks suggest experimenting with plant-based inks for digitally printing wafer electronic devices in cohesion with graphene and charcoal for enhanced functionality and sustainable output. Industrial applications of the research findings are recommended for the future for greater benefits. Lastly, the research investigations propel ‘Biophilic Colours’ (Rogers, n.d.); that is, life-loving colours from nature.

## Data Availability

Authors consent to the availability of data and materials.
